# Limited Weight Impact After Switching From Boosted Protease Inhibitors to Dolutegravir in Persons With Human Immunodeficiency Virus With High Cardiovascular Risk: A Post Hoc Analysis of the 96-Week NEAT-022 Randomized Trial

**DOI:** 10.1093/cid/ciac827

**Published:** 2023-03-04

**Authors:** Laura Waters, Lambert Assoumou, Ana González-Cordón, Stefano Rusconi, Pere Domingo, Mark Gompels, Stephane de Wit, François Raffi, Christoph Stephan, Mar Masiá, Jürgen Rockstroh, Christine Katlama, Georg M. N. Behrens, Graeme Moyle, Margaret Johnson, Julie Fox, Hans-Jürgen Stellbrink, Giovanni Guaraldi, Eric Florence, Stefan Esser, José M. Gatell, Anton Pozniak, Esteban Martínez, NEAT 022 study group investigators, Linos Vandekerckhove, Els Caluwé, Stephane de Wit, Coca Necsoi, Eric Florence, Maartje Van Frankenhuijsen, François Raffi, Clotilde Allavena, Véronique Reliquet, David Boutoille, Morane Cavellec, Elisabeth André-Garnier, Audrey Rodallec, Thierry Le Tourneau, Jérôme Connault, Jean-Michel Molina, Samuel Ferret, Miresta Previlon, Yazdan Yazdanpanah, Roland Landman, Véronique Joly, Adriana Pinto, Christine Katlama, Fabienne Caby, Nadine Ktorza, Luminita Schneider, Christoph Stephan, Timo Wolf, Gundolf Schüttfort, Juergen Rockstroh, Jan-Christian Wasmuth, Carolynne Schwarze-Zander, Christoph Boesecke, Hans-Jurgen Stellbrink, Christian Hoffmann, Michael Sabranski, Stephan Esser, Robert Jablonka, Heidi Wiehler, Georg M. N. Behrens, Matthias Stoll, Gerrit Ahrenstorf, Giovanni Guaraldi, Giulia Nardini, Barbara Beghetto, Antonella D’Arminio Montforte, Teresa Bini, Viola Cogliandro, Massimo Di Pietro, Francesco Maria Fusco, Massimo Galli, Stefano Rusconi, Andrea Giacomelli, Paola Meraviglia, Esteban Martinez, Ana González-Cordón, José Maria Gatell, Berta Torres, Pere Domingo, Gracia Mateo, Mar Gutierrez, Joaquin Portilla, Esperanza Merino, Sergio Reus, Vicente Boix, Mar Masia, Félix Gutiérrez, Sergio Padilla, Bonaventura Clotet, Eugenia Negredo, Anna Bonjoch, José L. Casado, Sara Bañón-Escandell, Jose Saban, Africa Duque, Daniel Podzamczer, Maria Saumoy, Laura Acerete, Juan Gonzalez-Garcia, José Ignacio Bernardino, José Ramón Arribas, Victor Hontañón, Graeme Moyle, Nicole Pagani, Margherita Bracchi, Jaime Vera, Amanda Clarke, Tanya Adams, Celia Richardson, Alan Winston, Borja Mora-Peris, Scott Mullaney, Laura Waters, Nahum de Esteban, Ana Milinkovic, Sarah Pett, Julie Fox, Juan Manuel Tiraboschi, Margaret Johnson, Mike Youle, Chloe Orkin, Simon Rackstraw, James Hand, Mark Gompels, Louise Jennings, Jane Nicholls, Sarah Johnston

**Affiliations:** Belgium; France; Germany; Italy; Spain; United Kingdom; 1 Mortimer Market Centre, Central and North West London National Health Service (NHS) Foundation Trust, London, United Kingdom; 2 Sorbonne Université, Inserm, Institut Pierre Louis d’Épidémiologie et de Santé Publique, Paris, France; 3 Hospital Clínic, Consorci Institut D’Investigacions Biomediques August Pi i Sunyer, Universitat de Barcelona; 4 Centro de Investigación Biomédica en Red de Enfermedades Infecciosas, Instituto de Salud Carlos III, Madrid, Spain; 5 Unità Operativa Malattie Infettive, Ospedale Civile di Legnano, Azienda Socio Sanitaria Territoriale Ovest Milanese, Legnano (MI), Italy; 6 Hospital de Sant Pau, Barcelona, Spain; 7 North Bristol NHS Trust, Bristol, United Kingdom; 8 Centre Hospitalier Universitaire Saint-Pierre, Brussels, Belgium; 9 Centre Hospitalier Universitaire, Nantes, France; 10 Universitätsklinikum, Goethe-University, Abteilung für Infektionskrankheiten, Frankfurt, Germany; 11 Hospital General Universitario de Elche, Spain; 12 Universitätsklinikum, Bonn, Germany; 13 Hôpital Universitaire Pitié Salpêtrière, Paris, France; 14 Medizinische Hochschule, Hannover, Germany; 15 Chelsea and Westminster Hospital NHS Foundation Trust; 16 Royal Free London NHS Foundation Trust; 17 Guy’s and St Thomas’ NHS Foundation Trust/ King’s College, London, United Kingdom; 18 Infektionsmedizinisches Centrum, Hamburg, Germany; 19 Università degli Studi di Modena e Reggio Emilia, Modena, Italy; 20 Instituut voor Tropische Geneeskunde, Antwerp, Belgium; 21 Universitätsklinikum, Essen, Germany; 22 ViiV Healthcare, Barcelona, Spain

**Keywords:** weight, switch, dolutegravir

## Abstract

**Background:**

In the NEAT022 trial, virologically suppressed persons with human immunodeficiency virus (HIV) at high cardiovascular risk switching from protease inhibitors to dolutegravir either immediately (DTG-I) or after 48 weeks (DTG-D) showed noninferior virological suppression and significant lipid and cardiovascular disease risk reductions on switching to dolutegravir relative to continuing protease inhibitors.

**Methods:**

In post hoc analysis, major endpoints were 48-week and 96-week weight and body mass index (BMI) changes. Factors associated with weight/BMI changes within the first 48 weeks of DTG exposure, proportion of participants by category of percentage weight change, proportions of BMI categories over time, and impact on metabolic outcomes were also assessed.

**Results:**

Between May 2014 and November 2015, 204 (DTG-I) and 208 (DTG-D) participants were included. Weight significantly increased (mean, +0.810 kg DTG-I arm, and +0.979 kg DTG-D arm) in the first 48 weeks postswitch, but remained stable from 48 to 96 weeks in DTG-I arm. Switching from darunavir, White race, total to high-density lipoprotein cholesterol ratio <3.7, and normal/underweight BMI were independently associated with higher weight/BMI gains. The proportion of participants with ≥5% weight change increased similarly in both arms over time. The proportions of BMI categories, use of lipid-lowering drugs, diabetes and/or use of antidiabetic agents, and hypertension and/or use of antihypertensive agents did not change within or between arms at 48 and 96 weeks.

**Conclusions:**

Switching from protease inhibitors to dolutegravir in persons with HIV with high cardiovascular risk led to modest weight gain limited to the first 48 weeks, which involved preferentially normal-weight or underweight persons and was not associated with negative metabolic outcomes.

**Clinical Trials Registration:**

NCT02098837 and EudraCT 2013-003704-39.

## Graphical abstract

**Graphical Abstract:**
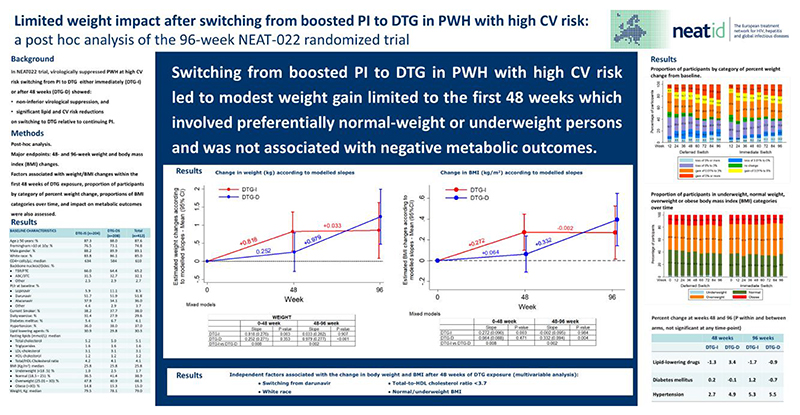

The European treatment network for HIV, hepatitis, and global infectious diseases (NEAT)–022 is a randomized, noninferiority, strategic trial comparing the efficacy, safety, and impact on plasma lipids of switching the boosted protease inhibitor (PI/r) component to dolutegravir (DTG) versus continuing PI/r in persons with human immunodeficiency virus (PWH) suppressed on 2 nucleoside reverse transcriptase inhibitors (NRTIs) plus a PI/r. Participants were considered at high risk for cardiovascular disease (CVD) risk as they were required to be aged ≥50 years and/or have a Framingham 10-year risk score >10% at 10 years. Eligible subjects were randomized to immediate or deferred (week 48) switch to DTG and followed up for 96 weeks. The primary results at 48 weeks [1] and the final results at 96 weeks [2] demonstrated noninferior maintained virological suppression and significant lipid improvements and reduction in CVD risk score on switch to DTG relative to continuing PI/r.

Over recent years there have been several analyses of observational cohorts and randomized controlled trials showing a distinctive impact in weight gain with different combinations of antiretroviral therapy (ART). Integrase inhibitors, in particular DTG and bictegravir, and tenofovir alafenamide (TAF) have been specifically associated with higher weight increases; women, Black individuals, and older people appear to be especially at risk of excessive weight gain [3–5]. Because both excessive and insufficient body mass index (BMI) are associated with negative outcomes in the general [6] and the PWH [7] populations, understanding the real impact of different antiretrovirals on weight and the risk factors and possible mechanisms for ART-related weight change is of crucial importance.

Many of the analyses demonstrating higher weight gains with integrase inhibitors have emerged from trials undertaken in treatment-naive PWH in which the comparator arm usually contained efavirenz, a drug that may prevent weight gain [4, 8, 9]. In fact, efavirenz rapid metabolizers, who have lower plasma efavirenz levels, gained the same amount of weight than PWH treated with dolutegravir plus the same nucleoside backbone in the ADVANCE trial [Randomised, Phase 3 Non-inferiority Study of DTG + TAF + FTC Compared With DTG + TDF + FTC and EFV + TDF + FTC in Patients Infected With HIV-1 Starting First-line Antiretroviral Therapy [WRHI 060 (ADVANCE)] (https://clinicaltrials.gov/ct2/show/NCT03122262)] [10]. Data from switching studies have been less clear because of differences in prior regimens or concomitant changes in nucleoside backbone drugs among other factors [11–15]. More advanced human immunodeficiency virus (HIV) status (ie, high plasma HIV RNA levels or low CD4 cell counts) has been consistently associated with higher weight increases after ART initiation [3, 4]. The “return-to-health” phenomenon, whereby weight increases after ART initiation, has been well characterized [16] and analyzing the impact of ART switch in individuals who are virally suppressed may reduce the potential confounding of this phenomenon. We therefore analyzed the impact of switching from the PI/r component to DTG on weight in NEAT022, thus providing an ideal scenario of a randomized clinical trial, involving a pure drug change, that was replicated, free of the confounding “return-to-health” phenomenon characteristic of treatment-naive individuals, and including a homogeneous population at high cardiovascular risk.

## Methods

### Participants

The NEAT022 trial was conducted in 32 clinical sites in 6 European countries. Participants were recruited between May 2014 and November 2015. Eligible persons were PWH aged ≥50 years, aged >18 years with a Framingham CVD risk score >10% at 10 years, or both. They also had to be on a triple antiretroviral regimen consisting of a PI/r (ritonavir-boosted lopinavir, darunavir, atazanavir, saquinavir, or fosamprenavir) plus 2 NRTIs and with plasma HIV RNA <50 copies/mL for at least the previous 6 consecutive months. PWH with prior evidence of viral resistance based on the presence of any major resistance-associated mutations to NRTI backbone were excluded, as were those with prior virological failure while on ART unless there was a documented lack of selection of resistance mutations. The protocol was approved by the ethics committees of all participating sites. All participants provided written informed consent. The study is registered at ClinicalTrials.gov (NCT02098837) and EudraCT (2013-003704-39).

### Randomization and Masking

Eligible participants were randomly assigned 1:1 in an open-label fashion to either switch the PI/r component to DTG continuing the same background NRTI (immediate switch [DTG-I]), or to continue PI/r-based ART for 48 weeks (delayed switch [DTG-D]), at which point all participants remaining on a PI/r switched to DTG out to week 96 of follow-up. Participants were assigned to treatment groups by computer-generated permuted blocks of 4 and stratified by country.

### Study Procedures

Participants attended for study visits at screening, baseline, then every 12 weeks for 96 weeks thereafter with an additional visit at week 4 or 52 in the DTG-I or DTG-D group, respectively. Each visit included general assessment of vital signs, adverse events (AEs), and blood samples for routine safety, fasting lipid, and immunovirological measurements. At each visit, participants were provided advice about smoking cessation, daily exercise, weight, diet and alcohol intake, and blood pressure control using a predefined written formulary. AIDS events and deaths, serious AEs, AEs grade 3 or above, AEs leading to modification of study drugs, all protocol discontinuations, and all protocol-defined episodes of virological failures required confirmation by an independent endpoint review committee, whose members were blinded to specific treatment regimens.

### Endpoints

The major endpoints of this post hoc analysis were the changes in weight (kg) and in BMI (kg/m^2^) at week 48 and 96. Factors associated with the evolution of BMI and weight within the first 48 weeks on DTG (DTG-I group [0–48 weeks] and DTG-D group [48–96 weeks]) were also assessed. We studied the proportions of underweight (BMI <18.5 kg/m^2^), normal (BMI 18.5–25.0 kg/m^2^), overweight (BMI 25.01–30.0 kg/m^2^), and obese (BMI >30.0 kg/m^2^) participants over time. Furthermore, we analyzed the magnitude of weight change by category over time defined by the proportion of participants experiencing at least 3% and 5% weight gain or loss as potential clinically meaningful cutoffs [17]. We also assessed the number of PWH receiving lipid-lowering agents, with diabetes and/or taking antidiabetic agents and with hypertension and/or taking antihypertensive agents in each arm at baseline, week 48, and week 96. Finally, we investigated whether there was a relationship between lipids and weight at baseline, and between the change in lipids and the change in weight at week 48 and week 96.

### Statistical Analyses

The study was powered for a noninferiority efficacy endpoint. All randomized participants who received at one time the study treatment were included in the present analysis. The changes in weight and BMI over time were compared within and between the groups using mixed models for repeated measures with random effects and spatial power covariance structure. Missing weight was not imputed. We used linear mixed models to account for all participants in the analyses. The models included group, time, and interaction between group and time. Time was chosen as continuous variable.

Univariable and multivariable analyses identified factors associated with the change in BMI and weight on DTG and considered age, Framingham score (≤15% vs >15%), sex, race, HIV acquisition mode, CD4 cell count, hepatitis C antibody status, duration of viral suppression, time on combination ART, NRTI backbone, PI/r at baseline, estimated glomerular filtration rate, and cardiovascular risk factors. Parameters with univariable *P* <.15 were retained for the multivariable analysis and multivariable analysis was adjusted for baseline BMI. As some of these variables had missing values, we used multiple imputation approach to impute missing values. Continuous variables were modeled as categorical variables using terciles.

The evolution of proportions of PWH by BMI categories and at least 3% or 5% weight change over time and the evolution of proportions receiving lipid-lowering agents, with diabetes and/ or taking antidiabetic agents, and with hypertension and/or taking antihypertensive agents were compared within and between the 2 groups using generalized estimation equation models with unstructured covariance matrix. The models included treatment group, time, and interaction between treatment group and time. Time was chosen as categorical variable. Correlations between lipids and weight were assessed using nonparametric Spearman correlation test.

Variables were summarized as proportions for categorical variables, median and interquartile range (IQR) for continuous baseline variables, and mean and standard error (SE) for BMI and weight at each time point. All *P* values are 2-sided with a significance level of 5%. Analysis used SAS statistical analysis software version 9.4 and IBM SPSS statistics version 24.

## Results

Between May 2014 and November 2015, 455 participants were screened and 415 randomized: 205 to switch to a DTG-based regimen (DTG-I arm) and 210 to continue their PI/r-based regimen (DTG-D arm); 412 PWH received at least 1 dose of study treatment (204 and 208 in the DTG-I and DTG-D arms, respectively). The study flowchart is shown in Supplementary Figure 1. Baseline characteristics were balanced between study groups including the duration of previous virological suppression, distribution of baseline PI/r, NRTI, and the percentage of participants receiving lipid-lowering agents (Table 1). Of note, most participants were aged >50 years (88%), male (89%), and White (85%). For the DTG-D group, characteristics at time of switch to DTG were roughly similar to study baseline. Baseline mean BMI was 26.2 kg/m^2^ (SE, 0.28) and 26.1 kg/m^2^ (SE, 0.28) in the DTG-I and DTG-D groups, respectively. Baseline mean weight was 79.5 kg (SE, 0.94) and 78.8 kg (SE, 0.95) in the DTG-I and DTG-D groups, respectively.

**Figure 1:**
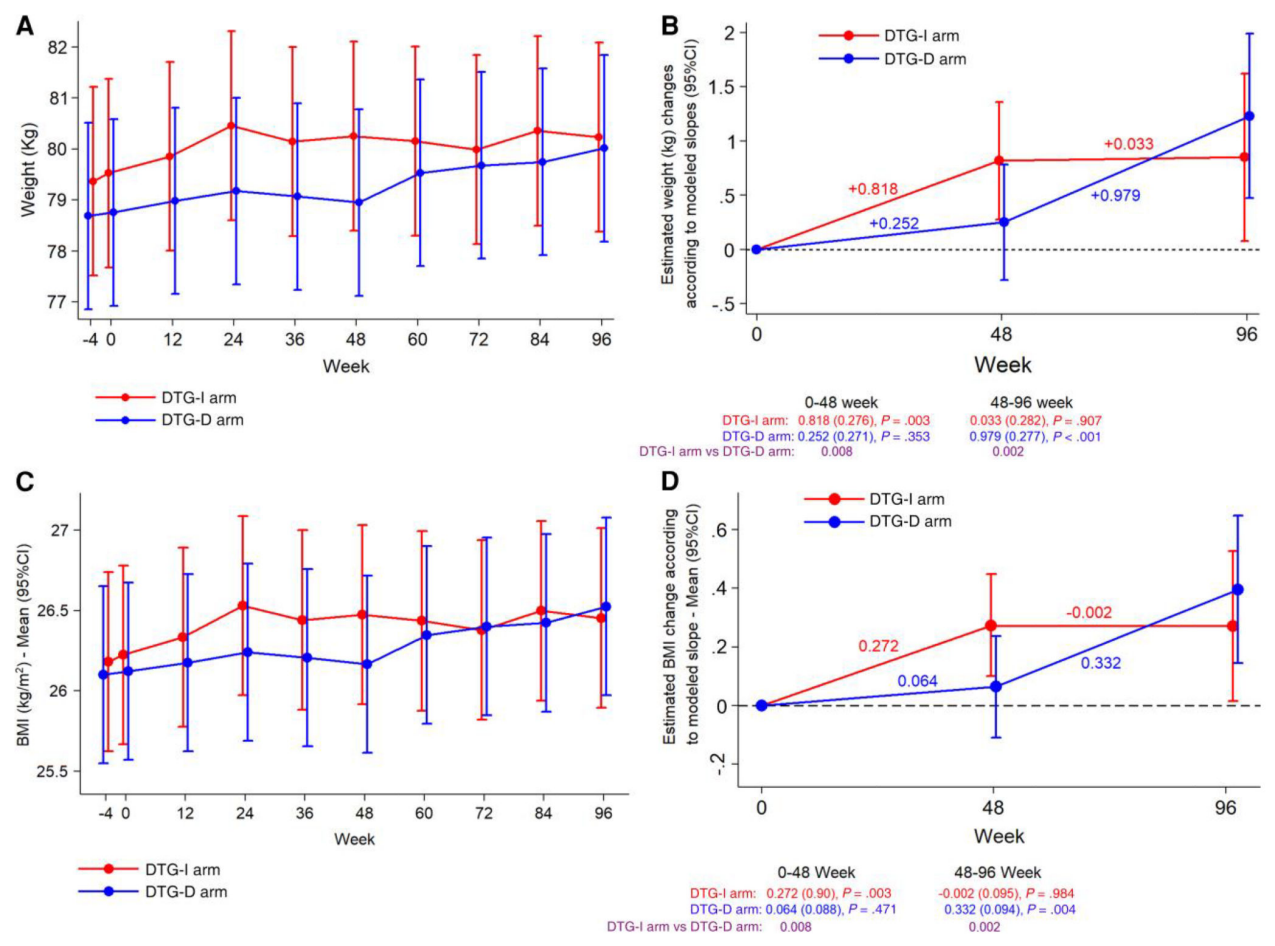
Evolution of weight and body mass index (BMI), and change in weight and BMI according to modeled slopes. *A*, Evolution of weight (kg). *B*, Change in weight (kg) according to modeled slopes. Comparison between dolutegravir immediate switch (DTG-I) and dolutegravir deferred switch (DTG-D) in weight gain from baseline to week 48 (*P*= .008) and from week 48 to week 96 (*P*= .002). *C*, Evolution of BMI (kg/m^2^). *D*, Change in BMI according to modeled slopes.

The evolution of weight and BMI over time in both arms and the slopes of weight and BMI changes over 96 weeks are shown in Figure 1, Supplementary Figure 2, and Supplementary Tables 1 and 2. The introduction of DTG increased weight and BMI, particularly during the first 24–48 weeks, and this finding was similarly reproduced in both arms. In the DTG-I arm in which the exposure to DTG was longer, weight and BMI remained stable after the initial 48-week gains.

During follow-up, 24 participants changed NRTI backbone for various reasons: 11 (5.4%) in the DTG-I arm and 13 (6.3%) in the DTG-D arm. Five persons in each arm received TAF during the study, none in the first 48 weeks. The change from baseline to week 96 in the percentage of persons actively smoking or doing daily exercise was −2.7% and 6.2% in the DTG-I arm and −4.3% and 6.7% in the DTG-D arm, respectively; none of these changes were statistically significant. The proportion of persons receiving lipid-lowering drugs changed −1.3% in DTG-I and 3.4% in DTG-D at 48 weeks and −1.7% in DTG-I and −0.9% in DTG-D at 96 weeks.

The proportion of persons with diabetes and/or taking antidiabetic agents changed −0.2% in DTG-I and −0.1% in DTG-D at 48 weeks and 1.2% in DTG-I and −0.7% in DTG-D at 96 weeks. The proportion of persons with hypertension and/or taking antihypertensive agents changed 2.7% in DTG-I and 4.9% in DTG-D at 48 weeks and 5.3% in DTG-I and 5.5% in DTG-D at 96 weeks. None of these comparisons within or between groups was significant.

Table 2 shows the univariable (full univariable analysis is shown in Supplementary Table 3) and multivariable analysis of factors associated with the change in body weight and BMI at week 48. Switching from boosted darunavir (vs other boosted PI), being White (vs other races), having a total to high-density lipoprotein (HDL) cholesterol ratio <3.7 (vs ≥3.7), and having a normal or underweight BMI (vs overweight or obese BMI) were independently associated with higher weight gains. Similar results were roughly reproduced when considering independent risk factors for higher BMI gains.

**Figure 2:**
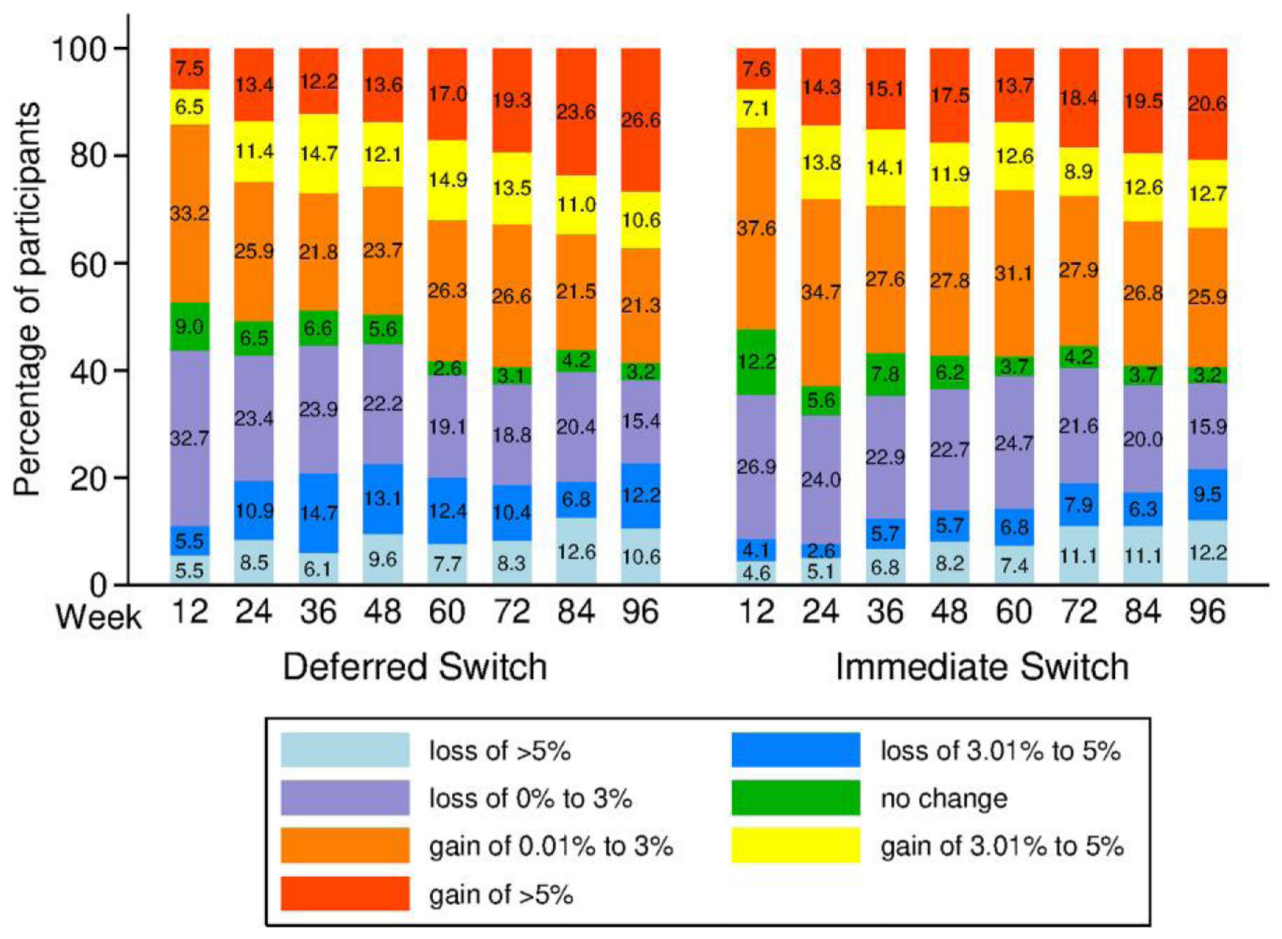
Evolution of the proportion of participants by category of percentage weight change from baseline.

**Figure 3:**
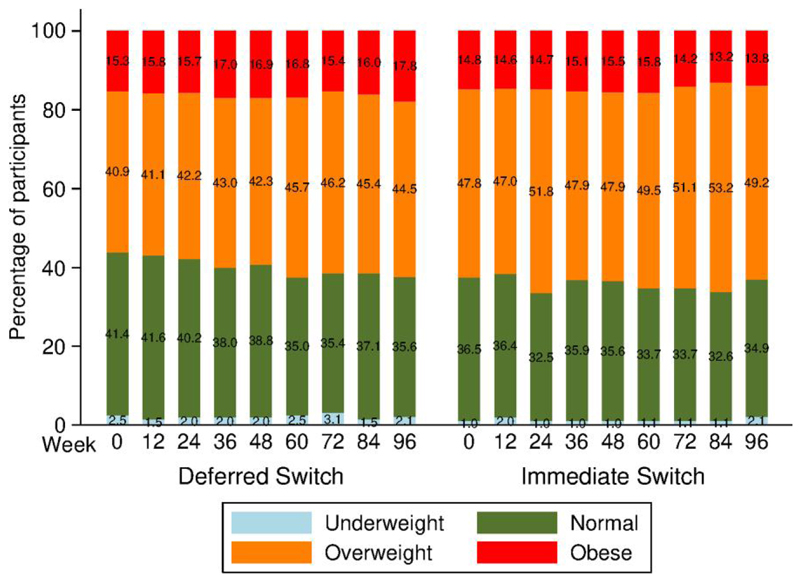
Proportion of participants in underweight, normal weight, overweight, or obese body mass index categories over time.

We analyzed the magnitude of weight gain by category over time. The proportions of individuals experiencing 0–3%, >3% to 5%, and >5% weight gain or loss are illustrated in Figure 2. The proportion of participants who gained at least 5% weight increased significantly from 7.6% at week 12 to 17.5% at week 48 in the DTG-I arm (*P*= .003) and nonsignificantly from 7.5% at week 12 to 13.6% at week 48 in the DTG-D arm (*P*= .064), with a difference at week 48 of −3.9% (95% CI: −11.1% to 3.3%) between the 2 groups, which was nonsignificant. The proportion of participants who gained at least 5% weight increased significantly in both arms from 7.6% at week 12 to 20.6% at week 96 (*P* < .001) in the DTG-I and from 7.5% at week 12 to 26.6% at week 96 (*P* < .001) in the DTG-D arm, with a nonsignificant difference at week 96 of −6.0% (95% CI: −14.6% to 2.6%) between groups. Similarly, the proportions of participants who lost at least 5% weight also increased significantly in each arm with no significant differences at week 96 between groups. Despite these significant changes in the extreme categories of at least 5% weight gain or loss, the proportions of individuals who were underweight, normal weight, overweight, or obese did not change significantly over time in each arm and the differences between arms at 48 and 96 weeks were not statistically significant either (Figure 3). Among PWH with normal BMI at baseline (n = 158), 17.1% in the DTG-I arm and 20.3% in the DTG-D arm became overweight (*P* = .887), and none became obese. There were weak correlations between weight and triglycerides at baseline (*R* = 0.15, *P* = .003). There were also weak correlations between changes in weight and changes in lipids at 48 (total cholesterol: *R* = 0.151, *P* = .003; low-density lipoprotein [LDL] cholesterol: *R* = 0.122, *P* = .016; and triglycerides: *R* = 0.150, *P* = .003) and 96 (total cholesterol: *R* = 0.127, *P* = .013; LDL cholesterol: *R* = 0.141, *P* = .016; and triglycerides: *R* = 0.192, *P* < .001) weeks.

## Discussion

We assessed the impact on weight of switching from PI/r to DTG in virologically suppressed PWH with high cardiovascular risk. The switching strategy was pure as the only antiretroviral change performed was the replacement of PI/r by DTG, while the nucleoside background remained unchanged. Switching from PI/r to DTG led to significant albeit numerically small weight gains in the first 48 weeks after the switch. The pattern was consistently found in both the DTG-I and DTG-D arms. Discontinuation of PI/r, introduction of DTG, or both could have been involved. Interestingly, in the DTG-I group, which was exposed to DTG in the trial for 96 weeks, there were no further weight changes between 48 and 96 weeks, suggesting that the initial weight gain impact associated with the switching strategy may not necessarily be sustained over time. The amount of weight gain in the first 48 weeks of DTG exposure was 0.818 kg (DTG-I arm) or 0.979 kg (DTG-D arm). As a reference, reported annual weight gain in European adult populations has been 0.3–0.5 kg [18].

We identified several independent risk factors at baseline associated with a higher weight increase after the first 48 weeks of DTG exposure. PWH switching from boosted darunavir experienced a higher weight gain than PWH switching from other PIs. In a Spanish multicenter randomized clinical trial comparing between ritonavir-boosted darunavir and boosted atazanavir plus tenofovir disoproxil fumarate (TDF)/emtricitabine in antiretroviral-naive PWH, darunavir showed a better lipid profile [19] and less fat gain and less insulin resistance [20] than atazanavir at 96 weeks, although a US trial with similar ART regimens and follow-up did not find such differences [21, 22]. Another explanation may be plausible. Ritonavir boosting increases tenofovir exposure when concomitantly administered with TDF [23], but the effect seems higher with darunavir [24] than with atazanavir [25]. As TDF suppresses weight gain [26], discontinuation of boosted darunavir might be associated with higher weight gain than discontinuation of boosted atazanavir.

In contrast to other studies, White race was associated with a higher weight gain although 85% of NEAT022 participants were of White race, making the study underpowered to examine an association between race and weight change. Weight was gained inversely to baseline BMI status, and it did not specially affect overweight or obese PWH. These findings suggest that the modest weight gain associated with switching from PI/r to DTG preferentially involved metabolically healthier people as reflected by their characteristics of normal (rather than elevated) total to HDL cholesterol ratio and underweight/normal (instead of overweight/obese) BMI among this population with high cardiovascular risk. It is reassuring that weight gain after switching from PI/r to DTG did not have an impact on those with a higher BMI or worse metabolic status. In a pooled analysis of 12 prospective clinical trials wherein virologically suppressed PWH were randomized to switch or remain on a stable baseline regimen, moderate weight gains after antiretroviral switch were common and usually plateaued by 48 weeks [27], findings similar to those in NEAT022. In such pooled analysis, weight gain was correlated more strongly with baseline regimen, especially switch off drugs preventing weight gain such as TDF or efavirenz, and with younger age and lower baseline BMI than with sex-, race-, or HIV-related factors.

There were significant changes (both increases and losses) in proportions of persons in the extreme categories of percentage weight change considered (>3% to 5%, and >5%) in both arms without significant differences at 96 weeks between arms, in accordance with the global changes in seen in weight and BMI. Exposure to DTG over 96 weeks did not increase further the proportions of persons who gained >3% to 5% or >5% of baseline weight relative to exposure to DTG over 48 weeks. The proportions of PWH according to BMI categories did not change significantly over time in each arm and the differences between arms at 48 and 96 weeks were not statistically significant either.

This study has limitations. We chose the 5% weight gain as a potential clinically meaningful cutoff because this threshold was associated with insulin resistance in a recent PWH cohort study [17], but there is no current consensus on which weight gain threshold is clinically meaningful. In the NEAT022 study, we did not assess insulin resistance but the switching strategy was not associated with worse metabolic outcomes. The 5% threshold is widely accepted for clinically significant weight loss as it has been associated with improved metabolic function and cardiovascular risk scores in people with obesity [28]. One important limitation of percentage weight change is that it depends on baseline weight [29]. Because weight gain in the NEAT022 study preferentially affected those with normal or underweight BMI, the proportion of persons exceeding 5% weight gain threshold might have been overrepresented. The specific characteristics of the population and type of ART replaced by DTG should be carefully considered before extrapolating these results to other populations or antiretroviral drugs switched. We did not collect information on food intake and physical exercise, but all participants received similar standardized lifestyle advice and the randomized nature of the study should not account for differences between arms. Finally, we did not undertake any anthropometric or body composition measurements to assess lean versus fat mass and subcutaneous versus visceral adiposity, all of which may influence the clinical impact of weight gain per se.

In conclusion, switching from PI/r to DTG in PWH with high cardiovascular risk led to modest weight gain limited to the first 48 weeks, which involved preferentially normal-weight or underweight persons and was not associated with negative metabolic outcomes. Further long follow-up studies are needed to see whether these findings are confirmed in other populations and with the use of other antiretroviral drugs.

## Supplementary Material

Supplementary data Supplementary materialsare available at Clinical Infectious Diseases online. Consisting of data provided by the authors to benefit the reader, the posted materials are not copyedited and are the sole responsibility of the authors, so questions or comments should be addressed to the corresponding author.

## Figures and Tables

**Table 1:** Baseline Characteristics

Characteristic	DTG-I (n = 204)	DTG-D (n = 208)	Total (N = 412)
Age, years, median (IQR)	54 (51–58)	53 (51–57)	54 (51–58)
Age ≥50 years	178 (87.3)	183 (88.0)	361 (87.6)
Framingham score at 10 years			
<10	48 (23.5)	56 (26.9)	104 (25.2)
10–15	61 (29.9)	53 (25.5)	114 (27.7)
15–20	43 (21.1)	51 (24.5)	94 (22.8)
>20	52 (25.4)	48 (23.1)	100 (24.3)
Male gender	180 (88.2)	187 (89.9)	367 (89.1)
White race	171 (83.8)	179 (86.1)	350 (85.0)
Mode of HIV-1 transmission			
Men who have sex with men	130 (63.7)	130 (62.5)	260 (63.1)
Heterosexual	49 (24.0)	47 (22.6)	96 (23.3)
Other	25 (12.3)	31 (14.9)	56 (13.6)
CD4^+^ count, cells/μL, median (IQR)	634 (488–819)	584 (470–839)	610 (476–830)
HIV RNA >50 copies/mL	7 (3.4)	1 (0.5)	8 (2.0)
Hepatitis C IgG antibodies detected	29 (14.4)	25 (12.1)	54 (13.2)
Time since undetectable viral load (<50 copies per mL), y, median (IQR)	5.5 (2.6–9.6)	5.7 (2.7–8.9)	5.7 (2.7–9.3)
Backbone nucleos(t)ides			
TDF/emtricitabine	134 (66.0)	134 (64.4)	268 (65.2)
Abacavir/lamivudine	64 (31.5)	68 (32.7)	132 (32.1)
Other	5 (2.5)	6 (2.9)	11 (2.7)
PI/r at baseline			
Lopinavir	12 (5.9)	23 (11.1)	35 (8.5)
Darunavir	105 (51.7)	108 (51.9)	213 (51.8)
Atazanavir	77 (37.9)	71 (34.1)	148 (36.0)
Other	9 (4.4)	6 (2.9)	15 (3.7)
Current smoker	78 (38.2)	78 (37.7)	156 (38.0)
Daily exercise	64 (31.4)	58 (27.9)	122 (29.6)
Diabetes mellitus and/or antidiabetic agents	11 (5.4)	14 (6.7)	25 (6.1)
Hypertension and/or antihypertensive agents	73 (36.0)	79 (38.0)	152 (37.0)
Family history of cardiovascular disease	87 (43.5)	89 (43.6)	176 (43.6)
Receiving lipid-lowering agents	63 (30.9)	62 (29.8)	125 (30.3)
Fasting plasma lipids, mmol/L, median (IQR)			
Total cholesterol	5.2 (4.4–5.8)	5.0 (4.5–5.6)	5.1 (4.5–5.7)
Triglycerides	1.6 (1.2–2.3)	1.6 (1.2–2.2)	1.6 (1.2–2.2)
Non–HDL-c	3.9 (3.3–4.6)	3.8 (3.2–4.3)	3.8 (3.2–4.5)
LDL-c	3.1 (2.5–3.7)	3.1 (2.5–3.6)	3.1 (2.5–3.6)
HDL-c	1.2 (1.0–1.5)	1.2 (1.0–1.4)	1.2 (1.0–1.4)
Total cholesterol/HDL-c	4.2 (3.4–5.4)	4.1 (3.4–5.2)	4.1 (3.4–5.3)
eGFR, mL/min, median (IQR)	91.0 (80.7–99.7)	91.4 (77.1–102.0)	91.2 (79.8–100.8)
BMI, kg/m^2^, median (IQR)	25.8 (23.6–28.0)	25.8 (23.5–28.2)	25.8 (23.5–28.2)
Underweight (<18.5)	2 (1.0)	5 (2.5)	7 (1.7)
Normal (18.5–25.0)	74 (36.5)	84 (41.4)	158 (38.9)
Overweight (25.01–30.0)	97 (47.8)	83 (40.9)	180 (44.3)
Obese (>30.0)	30 (14.8)	31 (15.3)	61 (15.0)
Weight, kg, median (IQR)	79.5 (72.1–86.0)	78.1 (69.5–87.8)	79.0 (71.0–87.0)

Data are presented as No. (%) unless otherwise indicated. Abbreviations: BMI, body mass index; DTG-D, dolutegravir deferred switch; DTG-I, dolutegravir immediate switch; eGFR, estimated glomerular filtration rate; HDL-c, high-density lipoprotein cholesterol; HIV, human immunodeficiency virus; IgG, immunoglobulin G; IQR, interquartile range; LDL-c, low-density lipoprotein cholesterol; PI/r, protease inhibitor with ritonavir; TDF, tenofovir disoproxil fumarate.

**Table 2:** Factors Associated With Change in Body Mass Index and Weight Within the First 48 Weeks on Dolutegravir (Immediate Switch Arm [0–48 Weeks] and Deferred Switch Arm [48–96 Weeks])

			Change From Baseline at Week 48			
			Univariable Analysis		Multivariable Analysis	
Characteristic	Parameter	Baseline Value Mean (SD)	Mean Gain (95% CI)	*P* Value	Mean Gain (95% CI)	*P* Value
Change in weight (kg)						
PI at baseline	Darunavir	79.6 (13.9)	1.335 (.815–1.855)	.0216	1.306 (.716–1.895)	.0261
	Atazanavir	80.0 (13.4)	0.291 (−.346 to .929)		0.304 (−.469 to 1.077)	
	Other (lopinavir, saquinavir, fosamprenavir)	77.2 (13.8)	0.374 (−.713 to 1.462)		0.317 (−.996 to 1.629)	
Race	White	79.4 (13.8)	1.005 (.616–1.395)	.1142	1.003 (.494–1.512)	.0370
	Black	80.2 (13.3)	−0.033 (−1.264 to 1.198)		−0.085 (−1.309 to 1.139)	
	Other	79.8 (13.2)	−0.227 (−1.822 to 1.368)		−0.351 (−2.418 to 1.715)	
Triglycerides at baseline, mmol/L	<1.3	75.6 (13.4)	1.477 (.853–2.101)	.0101	1.439 (.642–2.236)	.1161
	1.3–1.9	79.6 (12.4)	0.975 (.334–1.616)		0.976 (.278–1.673)	
	>1.9	83.1 (14.2)	0.138 (−.474 to .75)		0.102 (−.608 to .813)	
TC/HDL-c ratio at baseline	<3.7	75.2 (12.3)	1.623 (1.007–2.239)	.0099	1.615 (.795–2.434)	.0361
	3.7–4.8	80.3 (13.5)	0.371 (−.262 to 1.005)		0.345 (−.374 to 1.065)	
	>4.8	82.9 (14.2)	0.529 (−.093 to 1.152)		0.518 (−.152 to 1.188)	
Non–HDL-c at baseline, mmol/L	<3.4	78.7 (13.5)	1.255 (.635–1.874)	.1132	1.232 (.320–2.143)	.9517
	3.4–4.2	79.7 (13.8)	0.775 (.137–1.412)		0.774 (−.055 to 1.602)	
	>4.2	80.1 (13.9)	0.516 (−.111 to 1.143)		0.483 (−.232 to 1.197)	
BMI at baseline, kg/m^2^	Underweight (<18.5)	50.2 (5.2)	4.12 (1.081–7.158)	.0007	4.093 (2.771–5.415)	.0079
	Normal (18.5–25.0)	69.3 (8.9)	1.619 (.999–2.239)		1.599 (.962–2.236)	
	Overweight (25.01–30.0)	82.6 (7.9)	0.408 (−.157 to .974)		0.386 (−.212 to .985)	
	Obese (>30.0)	97.2 (12.2)	−0.012 (−.974 to .949)		−0.015 (−.813 to .784)	
Change in BMI (kg/m^2^)							
PI at baseline	Darunavir	26.4 (4.2)	0.462 (.286–.638)	.0154	0.453 (.261–.646)	.0158
	Atazanavir	26.4 (4.1)	0.098 (−.118 to .314)		0.102 (−.152 to .355)	
	Other (lopinavir, saquinavir, fosamprenavir)	25.4 (3.8)	0.106 (−.267 to .478)		0.079 (−.344 to .502)	
Race	White	26.1 (4.1)	0.341 (.209–.473)	.1424	0.339 (.170–.509)	.0420
	Black	27.4 (3.7)	0.014 (−.403 to .432)		−0.001 (−.404 to .402)	
	Other	26.7 (3.7)	−0.064 (−.605 to .477)		−0.124 (−.824 to .575)	
Triglycerides at baseline, mmol/L	<1.3	25.3 (3.9)	0.512 (.301–.724)	.0102	0.499 (.239–.759)	.1046
	1.3–1.9	26.3 (4.1)	0.317 (.099–.535)		0.314 (.082–.546)	
	>1.9	27.2 (4.1)	0.056 (−.152 to .263)		0.044 (−.195 to .283)	
TC/HDL-c ratio at baseline	<3.7	25.4 (4.1)	0.537 (.327–.747)	.0179	0.526 (.250–.801)	.0563
	3.7–4.8	26.2 (3.9)	0.141 (−.074 to .356)		0.137 (−.107 to .382)	
	>4.8	27.2 (4)	0.188 (−.023 to .399)		0.182 (−.047 to .411)	
BMI at baseline, kg/m^2^	Underweight (<18.5)	16.8 (0.8)	1.316 (.284–2.348)	.0009	1.296 (.802–1.790)	.0074
	Normal (18.5–25.0)	22.8 (1.6)	0.556 (.347–.764)		0.548 (.335–.762)	
	Overweight (25.01–30.0)	27 (1.3)	0.13 (−.062 to .322)		0.124 (−.074 to .322)	
	Obese (>30.0)	33.2 (2.6)	0.018 (−.305 to .341)		0.018 (−.247 to .283)	

Abbreviations: BMI, body mass index; CI, confidence interval; HDL-c, high-density lipoprotein cholesterol; PI, protease inhibitor; SD, standard deviation; TC, total cholesterol.
