# Supplementary figures and images for: Limited Weight Impact After Switching From Boosted Protease Inhibitors to Dolutegravir in Persons With Human Immunodeficiency Virus With High Cardiovascular Risk: A Post Hoc Analysis of the 96-Week NEAT-022 Randomized Trial
Source: Clin Infect Dis. Author manuscript; Available in PMC 2025 Feb 25. (PMC7617433; doi:10.1093/cid/ciac827)

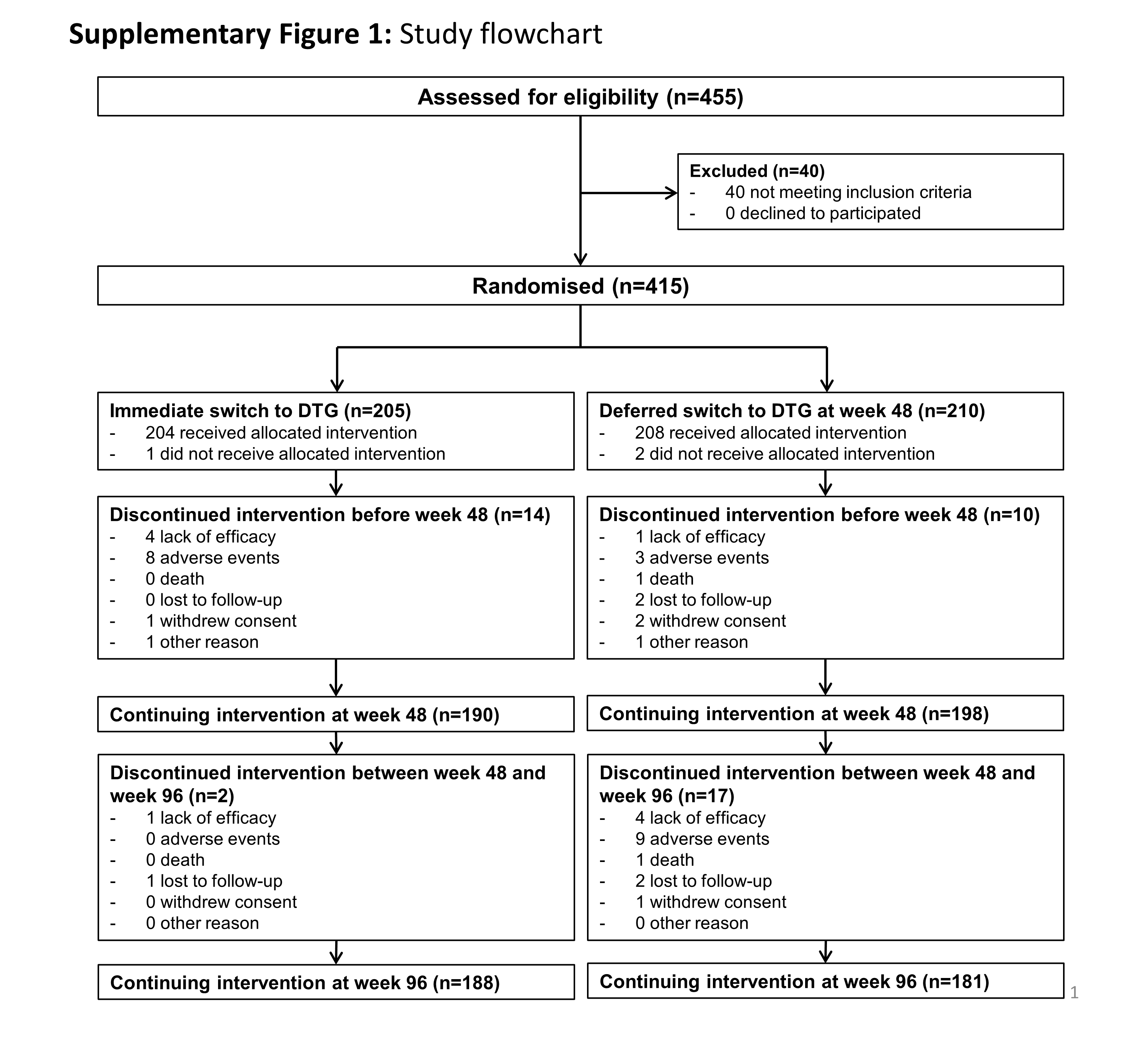

Supplement: Supplementary data — Supplementary materialsare available at Clinical Infectious Diseases online. Consisting of data provided by the authors to benefit the reader, the posted materials are not copyedited and are the sole responsibility of the authors, so questions or comments should be addressed to the corresponding author. [file EMS202957-supplement-Supplementary_data.zip › Supplementary Figure 1.tif]

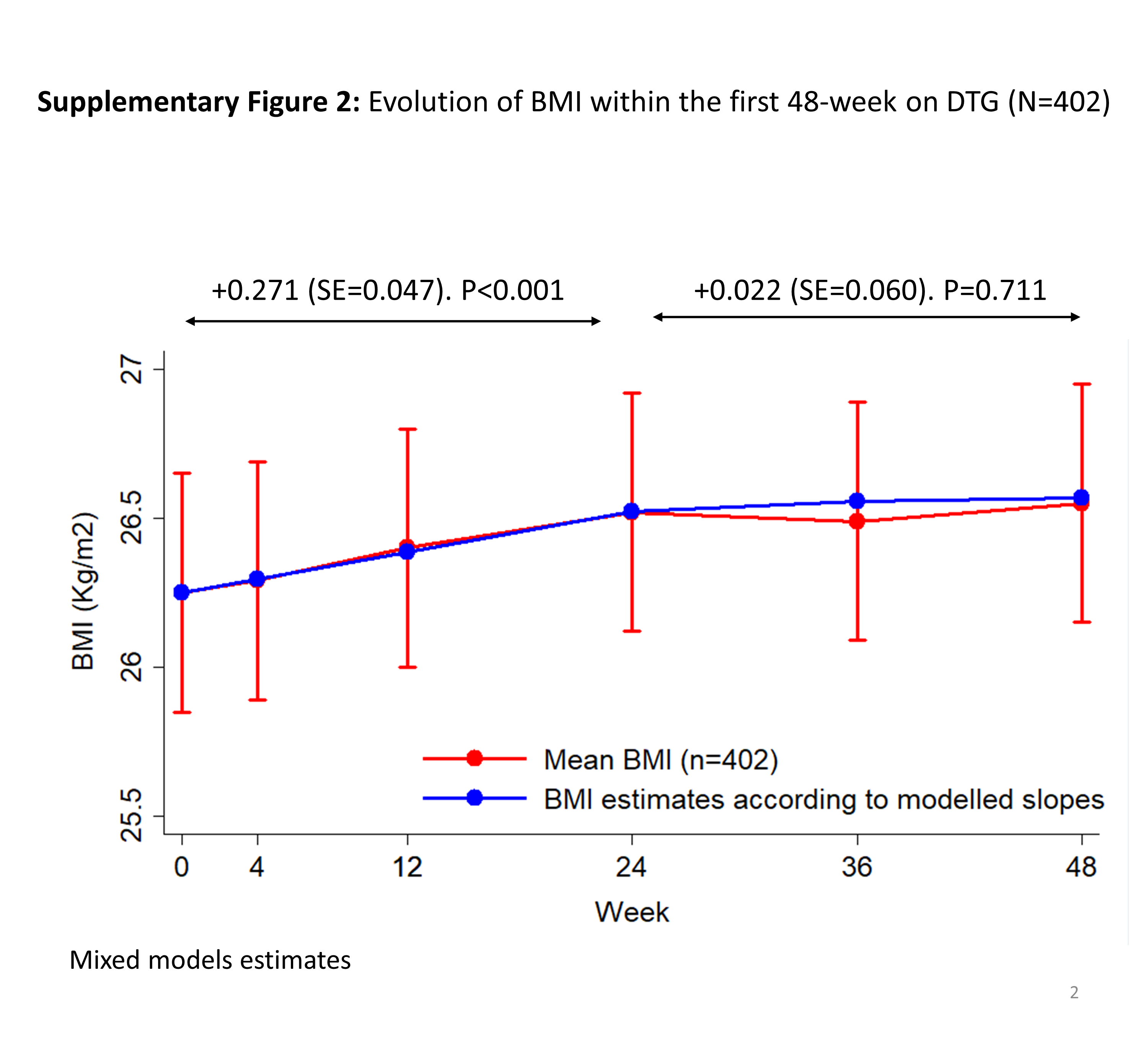

Supplement: Supplementary data — Supplementary materialsare available at Clinical Infectious Diseases online. Consisting of data provided by the authors to benefit the reader, the posted materials are not copyedited and are the sole responsibility of the authors, so questions or comments should be addressed to the corresponding author. [file EMS202957-supplement-Supplementary_data.zip › Supplementary Figure 2.tif]
